# Physiology and Treatment of Dental Pulp

**Published:** 1877-12

**Authors:** J. A. Watling


### PHYSIOLOGY AND TREATMENT OF DENTAL PULP.

BY PROF, J. A. WATLING.

*Read before the Michigan Dental Society.*

If we lay open a tooth, there is revealed to our vision, a soft exquisitely sensitive, vascular and nervous tissue, the dental pulp, which is continuous with the dental nerve and appendages.

The microscope reveals the presence of veins, arteries and nerves of a highly vascular character. The pulp is abundantly supplied with blood vessels, given off from the dental artery which enters at the apex of the tooth; the ramifications of these vessels are evenly distributed through the entire pulp substance.

*For further discussion of this topic see Tomes, Salter, Weil and Forget.

From this pulp is deposited the tooth substance, which goes to make the perfect tooth, and in fact it never ceases to deposit tooth substance through life, though after the eruption of the teeth, the process is much more gradual, yet it is steadily retreating and the pulp cavity is becoming smaller and smaller, until in very advanced age in some of the teeth, there is only a trace of it left. But the deposit of recent date, or that after the perfect development of the teeth, is of a different character from the tooth, it being of a more dense structure, and having a more transparent appearance. It can be very readily traced in the tooth with the naked eye, and under the microscope it has more the appearance of cementum. These examples are very nicely shown in the front teeth of persons of quite advanced age, where by *friction*, the teeth have worn away, revealing the well defined pulp cavity, perfectly filled up with ossified matter. No doubt exists, but that nature makes an effort to repair all injuries done to the human economy, and in cases of slow decay, there is a constant effort, on the part of the pulp, to repair the injury steadily going on, which is communicated to it by the nerve communication, through the dental fibrils. We see examples of this in what is termed Pulp Stone—the pulp receiving a warning that some destructive agent has attacked its fortress, makes an effort to protect itself,—but it has failed to make its deposit in the proper place,—it has become charged with lime salts, and failing to lodge them against the walls of the pulp cavity, finally they become crystalized in the body of the pulp, and we have the well known pulp stone, which is commonly found in teeth of slow decay. In such an instance nature has made a failure, to accomplish any good results, in her efforts to arrest the destruction of the tooth, though I am of the opinion that teeth with these deposits, are very much more dense in their structure, than are those in which it is absent. Whether this is the result of an infiltration of lime salts, through the dental tubes into the dentine, or whether these teeth have always been more dense, I am unable to say. That the pulp plays an important part through life in the economy of the tooth, it would seem hardly possible for any one to doubt, if its functions were ended with the eruption of the tooth, we should not see those deposits of osseous matter in the teeth, or any effort to repair the damaged structure but on the contrary, as nature abhors a vacnum, so she does any useless appendages, and the pulp, if it were not of importance, would not be there. I am aware that it has been claimed by some, that it does not play any very great part, in the welfare of the tooth, after it has been fully developed, and that it can be as successfully preserved by filling after the pulp has been extirpated. In this I must take a decided issue, and after years of close observation, I am led to believe that only in very few cases, is it necessary to destroy the pulp, and never is it excusable until every means has been exhausted in the trial for its restoration to health. It has been claimed that climatic influences promote or retard the successful treatment of the pulp; of this there is but little doubt, for we see good operators complain that they can save but a small percentage of the pulps that are exposed. The fine bracing climate of Michigan I think particularly favorable for this operation, as a failure is of rare occurence. I have so much confidence in the operation of capping the pulp, that it is very rarely that I resort to the extreme measure of extirpation. It has been said that our efforts should be more directed to the preservation of the teeth, than to the pulp; this sounds very well in theory, but in practice there is something wanting, and that is *life* in the tooth. That in many cases as a last resort, teeth have been preserved for many years, without pulps, all are well aware. But in how many cases, the most skillful operator, after performing a very tedious operation, one he may think to be among his best efforts, and the patient highly gratified, pays a large fee, sees the tooth in two or three years begin to disintegrate and crumble away, leaving the gold standing supported from the pulp cavity, in the roots, a monument of misplaced confidence in the destruction of the pulp. This has been my experience, especially in the molar teeth.

The ratio of teeth saved by the methods of capping, or extirpation, has been in the proportion of 10 of the former to 1 of the latter. It may be that I have been more ambitious to save the tooth with the pulp alive, than I have been after its extripation. I have too often experienced, that entire want of power, to preserve or prevent a tooth from going to pieces. I would not have you understand, by any means, that the destruction of the pulp always leads to such disastrous results; on the contrary I can recall a number of cases, that have stood for many years and are still good. I have only drawn the comparison. It is nearly fifty years, since the operation of capping an exposed pulp, was first suggested by Dr. Kocker, who thought he could save five out of six teeth so treated. If he was able to save 5-6 of the teeth, treated with exposed pulp, what ought we to do at the present time, with the increased knowledge, and the advantages of instruments, apparatus and materials? I don't think I over estimate when I say, that 49 out of 50 can be thus saved. A very great variety of materials has been recommended as proper substances, to be brought in contact with the pulp; or to be used in capping—among them we find lead, horn, gutta-percha, vulcanized rubber, plaster of paris, lacto-phosphate of lime, the various preparations of oxychloride of zinc, oxide of zinc, and creosote, all having their strong advocates, and all meeting with more or less success. There are some properties, that are requisite in a material, used for capping a pulp. It should be free from all irritating influence. It should be a non con-ductor of thermal changes. It should have adaptation, so that it may be brought in immediate contact with the pulp without pressure, and at the same time so intimately, that there will be no space intervening. In the various preparations of zinc, and the lacto-phosphate of lime, we find more of these properties than in any other substances. The original introducer of the lacto-phosphate of lime, has made wonderful claims to this material as a bone producer or pabulum, that the pulp may feed on and produce new dentine at its leisure, I can hardly indorse such an idea—it don't seem possible, for nature to appropriate from an an external source, that which should come through a natural channel. I should just as soon think of seeing a piece of beef steak converted into new muscle, by being applied to a wound on my arm, as to see the pulps of a tooth eating away at lacto-phosphate of lime, and converting it into new dentine. I do not question its being good material to be brought in contact with the pulp, and no doubt new dentine may form under it. I

only question the idea of the pulp taking any of the bone forming material, from the capping and converting it into new tooth substance. Of the creosote and oxide of zinc—it has many warm advocates,—I think is in more general use at the present time than any other material, and is very successfully employed. The method of using it is to mix up the dry oxide of zinc with creosote, to a paste, which is placed in direct contact with the pulp and over this is placed a covering of the oxychloride of zinc, which is in turn covered with metallic filling. I have practiced this method with success, but have found the following method quite as successful and a little easier to manipulate. The cavity being properly prepared, the cavity is moistened slightly with creosote. Then I cut a piece of fine silk court plaster just large enough to cover the bottom of the cavity, this is slightly moistened so it will adhere to the tooth, and with this the pulp is covered over, and the cavity is filled with oxychloride of zinc, which is cut away to form the proper cavity, and filled with gold; this may be done at one sitting, or the filling of gold may be deferred to some future time.

I don't lay any special claim to the medicinal properties of the court plaster, any farther than it is quite as acceptable to a wound as any substance that can be brought in contact with it, and its adhesive property renders it very adaptable to the irregular form of the cavity. It may be brought in direct contact with the pulp, without undue pressure, and at the same time protect it from the escharotic effects of the chloride of zinc. There will be little or no pain, if the oxychloride of zinc is of the same temperature as the tooth.

I am not positive there is always new dentine deposited under such fillings, I have very seldom taken the opportunity of investigating after the tooth has been filled. I think it is a matter of very little importance, so long as the tooth remains good, whether the pulp is protected with new bone, or an artificial covering. That the pulp does remain healthy for years under this treatment, I do know from large experience and actual observation.

